# Humoral and Cellular Defense Mechanisms in Rebel Workers of *Apis mellifera*

**DOI:** 10.3390/biology10111146

**Published:** 2021-11-06

**Authors:** Aneta Strachecka, Paweł Migdał, Karolina Kuszewska, Patrycja Skowronek, Marcin Grabowski, Jerzy Paleolog, Michał Woyciechowski

**Affiliations:** 1 Department of Invertebrate Ecophysiology and Experimental Biology, University of Life Sciences in Lublin, 20-950 Lublin, Poland; patrycja.skowronek@up.lublin.pl (P.S.); grabowski.entomologist@gmail.com (M.G.); jerzy.paleolog@up.lublin.pl (J.P.); 2 Department of Environment, Hygiene and Animal Welfare, Wroclaw University of Environmental and Life Sciences, 50-375 Wroclaw, Poland; pawel.migdal@upwr.edu.pl; 3 Institute of Environmental Sciences, Jagiellonian University, 30-387 Krakow, Poland; k.kuszewska@gmail.com (K.K.); michal.woyciechowski@uj.edu.pl (M.W.)

**Keywords:** fat body, hemocytes, honeybee, immunity, juvenile hormone, rebels, oenocytes, vitellogenin

## Abstract

**Simple Summary:**

Immune mechanisms in insects include cellular and humoral defenses. One of these cellular defense mechanisms is phagocytosis. The humoral defense components are produced in the fat body cells, the development of which depends on the juvenile hormone (JH) titers and vitellogenin (Vg) concentration. These mechanisms also regulate caste formation. In a colony, a queen, a worker, or a rebel can develop from eggs of the same genome. Rebels are reproductive workers that can lay eggs while maintaining tasks inside and outside the colony the same as sterile workers. The aim of our studies was to determine the phagocytic index, JH titer, Vg concentration, the number of oenocytes (fat body cells), and their size in the rebels and to compare them to those in normal workers. The rebels were characterized by high phagocytic indices, JH and Vg levels, and increased numbers and sizes of oenocytes in the fat body cells in comparison to the normal workers. These characteristics can be viewed as an evolutionary adaptation of these reproductive workers to life in a eusocial society.

**Abstract:**

The physiological state of an insect depends on efficiently functioning immune mechanisms such as cellular and humoral defenses. However, compounds participating in these mechanisms also regulate reproductive caste formation and are responsible for reproductive division of labor as well as for labor division in sterile workers. Divergent reaction of the same genotype yielding reproductive queens and worker castes led to shaping of the physiological and behavioral plasticity of sterile or reproductive workers. Rebels that can lay eggs while maintaining tasks inside and outside the colony exhibit both queen and worker traits. So, we expected that the phagocytic index, JH3 titer, and Vg concentration would be higher in rebels than in normal workers and would increase with their age. We also assumed that the numbers of oenocytes and their sizes would be greater in rebels than in normal workers. The rebels and the normal workers were collected at the age of 1, 7, 14, and 21 days, respectively. Hemolymph and fat bodies were collected for biochemical and morphological analyses. The high levels of JH, Vg, and the phagocytic index, as well as increased numbers and sizes of oenocytes in the fat body cells demonstrate the physiological and phenotypic adaptation of rebels to the eusocial life of honeybees.

## 1. Introduction

Fat body cells and hemocytes play a crucial role in insect immunity. Most proteins from hemolymph are produced in fat body cells, which also secrete antimicrobial peptides and constitute an important component of the humoral defense. In contrast, hemocytes are involved in cellular defense mechanisms, such as phagocytosis and wound repair [1,2]. Recent studies have shown that hemocytes are divided into those that circulate in the insect’s hemolymph and those that do not circulate, the so-called sessile hemocytes [3]. These concentrate on the outer surface of the dorsal vessel and, mainly, in specific foci in the heart; they are typically connected to abdominal segments [3]. As a result, the fat body has a segmental character and functions as a multifunctional system made up of many organs/segments where each segment works separately and they all contribute to a common metabolism [4]. This segmental nature of the two cooperating tissues/cell types (hemocytes in hemolymph and fat body cells) is crucial in the cascading processes related to immunity and aging of honeybee workers. The development of oenocytes in the fat body depends on juvenile hormone (JH) titers [5] that are different in various castes of females in eusocial haplodiploids [6]. According to Robinson et al. [7], the JH titer increases with age in the workers, while that of ecdysteroids decreases. Ecdysteroids act on vitellogenin (Vg; a zinc-binding glycolipoprotein) uptake from the hemolymph [8], therefore its levels are reduced with age [9]. Vg is synthesized in the trophocytes [10] (most likely in the third tergite of the fat body) and is immediately directed to the appropriate tissues by the circulatory system [4]. The changes of these hormones as a result of aging induce hemocyte death [6]. The dramatic loss of hemocytes through nuclear pycnosis at its transition to foraging is mediated through programmed cell-death and discloses the full regulatory plasticity of honeybee immunosenescence [6,11,12]. High JH titers of nurses influence the hypopharyngeal glands (HG) development [13] and high levels of abdominal lipids [14] in comparison to foragers. HG degradation is a result of hormone changes, the level of which depends on the rate of lipolysis in the fat body [15,16]. Well-developed HGs are characteristic for sterile workers which have a developmental cycle closely related to the labor division [17]. Queens have a reduced HG [18]. In emerging virgin queens, both JH and Vg hemolymph titers are elevated and remain high during aging [9].

Not only queens but also some workers can activate their ovaries. Such workers lay unfertilized eggs that develop into drones. The reproductive potential of individuals can be easily assessed by counting the ovariole number in the ovaries [19]. This number is determined during larval development and depends on quantity and quality of food [13], presence of a queen [20], and her mandibular gland pheromones [21]. Thus, workers developing at larval stage in a queenless colony become relatively selfish individuals with higher reproductive potential than those that develop in a colony with a queen. These workers called rebel workers are characterized not only by their increased reproductive potential but also other altered anatomical features (e.g., less developed HGs, larger mandibular glands; [20]), shortened preimaginal development [22], prolonged life [23], different foraging preferences [24], tendency to drift to other colonies [25], increased sucrose sensitivity [26], energy reserves and protein concentrations in fat body [4], and increased learning ability [27].

Since these rebels are so different from the other workers, but also queenlike in many respects, we expected that: (1) the phagocytic index would be higher in rebels, and would increase with their age quicker than in normal workers; (2) the titer of JH3 would be higher in rebels than in normal workers and would increase with their age; (3) Vg concentration in rebels would be high and would increase with their age as in queens, and unlike in normal workers; and (4) the numbers of oenocytes and their sizes would be greater in rebels than in normal workers. The aim of our studies was to determine the phagocytic index, JH titer, Vg concentration, the number of oenocytes (fat body cells), and their size in rebels as well as in normal workers at different ages.

## 2. Materials and Methods

This study was performed in June and July 2018 at the apiary of the University of Life Sciences in Lublin, Poland (51.224039 N—22.634649 E). We used three colonies of *Apis mellifera* honeybees.

### 2.1. Experimental Design

A queen was caged within a queen excluder comb-cage containing two empty combs (C1 and C2) for egg laying for 24 h, in each of the three unrelated source colonies, populating two-box hives (Dadant Blatt from Łysoń Beekeeping Company, Klecza Górna, Poland; 20 frames; 435 × 150 mm^2^). On the fifth day after the queens were released, every source colony was divided into two equal parts, each in a separate box according to Woyciechowski and Kuszewska’s [20] procedure. The first part (top box), containing the queen, workers, brood, and C1, was used for rearing normal (non-rebel) workers, whereas the other part (bottom box), without a queen but with workers, brood, and C2 served for rearing rebels. After sealing the larval cells in C1 and C2, the two boxes were put together again, respectively, to restore each of the three source colonies. After 18 days, the brood combs C1 and C2 were placed in an incubator (temperature of 34.5 °C, relative humidity of 60%). The emerging workers were captured, individually marked, and restored to their original source colonies. Some of them were not restored to the hives but allocated for biochemical analyses when they were one-day-old. Next, from each of the original colonies, 10 marked rebels and 10 normal workers, were collected after the workers reached ages of 7, 14, and 21 days, respectively. This provided the following database: 3 colonies × 2 phenotypes (rebels and normal workers) × 4 samplings × 10 individuals = a total of 240 workers.

### 2.2. Hemolymph Collection

A glass capillary (20 µL; ‘end to end’ type; without anticoagulant; Medlab Products, Raszyn, Poland) was individually inserted between the third and fourth tergite of living workers to obtain fresh hemolymph, according to Łoś and Strachecka’s [28] method. Hemolymph volumes were separately measured in each capillary. Hemolymph from one bee was collected into one sterile Eppendorf tube containing 10 µL of ice-cooled 0.6% NaCl. 10 µL of hemolymph solution (freshly collected) were used for phagocytic index determination, and the remaining volume was immediately refrigerated at −80 °C for further biochemical analyses.

### 2.3. Biochemical Analysis

The following parameters were determined in the hemolymph samples:

− Phagocytic index, according to Watson et al. [1] modified by Keehnen et al. [2] method. To investigate the presence of phagocytic cells, a 10 µL suspension of heat-killed *Escherichia coli* (Sigma-Aldrich, Poznań, Poland; *E. coli* K-12 strain) in Luria–Bertani medium (A&A Biotechnology, Gdańsk, Poland) was added to 10 uL fresh hemolymph. The solution was incubated for 30 min at room temperature and gently shaking every 10 min. For each sample, a 30 µL drop of the solution was placed on a microscope slide and left to adhere for 20 min in a humid chamber at room temperature (RT). Next, the slide was fixed in 96% methanol for 5 min and 10 µL freshly prepared 0, 4% trypan blue was added. The preparation, after 1–2 min incubation and drying, was imaged under immersion under a light microscope with a magnification of 1250× and the bacterial cells absorbed by 50 phagocytes were counted. From these data, the mean number of bacteria phagocytized by one hemocyte was calculated.
− JH3 titers, using Teal et al. [29] method: GC–MS analysis was carried out using a Shimadzu chromatograph coupled with a mass detector (GC MS QP-2010 Ultra EI NCI; Duisburg, Germany). The GC was equipped with both split/splitless injectors. The injection volume was 2 µL. Zebron ZB-5MSi column capillary 30 m × 0.25 mm × 0.25 was used. Temperature program from 50 °C to 260 °C in increments of 10 °C / min was used. The analysis was performed in the SIM mode (main ion 235 and additional ions 217, 189, 147, 125, 111). Isobutane was used for chemical ionization.
− 135 µL of methanol and 100 µL of hexane were added to 15 µL of hemolymph. The solution was vortexed at 3200 rpm for 2 min. The emulsion was broken by centrifugation at 10,000× *g* for 5 min and the organic layer was removed with a 250 µL syringe. The aqueous layer was extracted an additional two times as above with 100 µL hexane. The JH3 content was determined by comparing the sample measurement result to the standard curve, which was prepared from the JH3 standard (Sigma-Aldrich, Poznań, Poland; J2000-10MG) in the concentration range 1–10 ng/mL.
− Vg levels, using a Honey Bee Vitellogenin (VG) ELISA Kit (MyBioSource, Warsaw, Poland; MBS109137), according to the instructions attached to the test. This commercially available ELISA kit measures the Vg content in body fluids, tissue homogenates, secretions, or honey bee feces. The sensitivity of the test is 5 ng/mL. The measuring range is 31.2 ng/mL–1000 ng/mL (1.56–50 ng).

### 2.4. Fat Body Analysis

Immediately after collecting the hemolymph, the fat bodies from sternites and from the third, fourth, fifth, sixth, and seventh tergites (see Carreck et al. [30]) were dissected under a Stereo Zoom Microscope. The fat bodies from each segment were placed on glass slides in 0.6% sodium chloride (for injection) and covered with cover glasses. Microscopic preparations were observed, and the fat body cells were photographed with an Olympus DP72 Camera (Olympus BX61 Microscope; 20× magnification) with the DIC attachment. In each image, an area of 800 × 600 µm was marked out and the oenocytes’ number was counted. The length and width of the oenocytes were then measured at 40× magnification.

### 2.5. Anatomical Characteristics

In order to confirm whether the emerging bees were rebels or normal workers the number of ovarioles (ovarian tubules) was determined according to Woyciechowski and Kuszewska’s [20] method. The total number of ovarioles in both ovaries of each individual was counted. All ovarioles were stained with the Giemsa reagent (Sigma-Aldrich, Poznań, Poland) for 10 s before being measured.

### 2.6. Statistical Analysis

Data were analyzed using Statistica formulas (TIBCO Software, Palo Alto, CA, USA), 13.3 (2017) version for Windows—StatSoft Inc., Tulsa, OK, USA. To compare the number of ovarioles as well as morphological and biochemical parameters between the rebels and normal workers, a mixed model two-way and three-way ANOVA was used. The experimental group was a fixed effect, and the colony was a random effect. If a difference among the groups was statistically significant, the ANOVA procedure was followed with a multiple comparison testing using the post hoc Tukey HSD test.

## 3. Results

**Figure 1:**
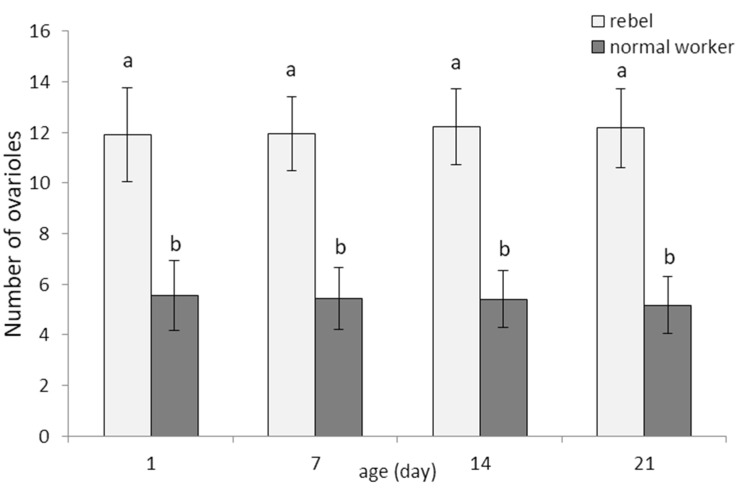
Number of ovarioles (mean ± SD) in normal workers and rebels. Lowercase letters indicate significant differences between averages for the rebels and normal workers within a specific age with *p* < 0.01; No. of rebels = 60; no. of normal workers = 60.

Regardless of age, the workers reared without the queen had a higher number of ovarioles than the workers reared in the queens’ presence (Figure 1). This result allowed us to continue research and compare the parameters of these two sub-castes of workers. Two-way ANOVA with multiple comparison testing using the post hoc Tukey HSD test; age F_(3,232)_= 0.13360, *p* = 0.93997; castes/sub-caste F_(1,2320)_= 1318.0; *p* < 0.0001, post-hoc for each group max. *p* = 0.999906; castes/sub-caste*age F_(3,232)_= 0.63239, *p* = 0.59480, post-hoc for each group max. *p* = 1.00; no. of rebels = 60; no. of normal workers = 60). Thus, we confirmed that the workers tested belonged to two different sub-castes, the rebels, and normal workers. This result allowed us to continue research and compare the parameters of these two sub-castes of workers.

**Figure 2:**
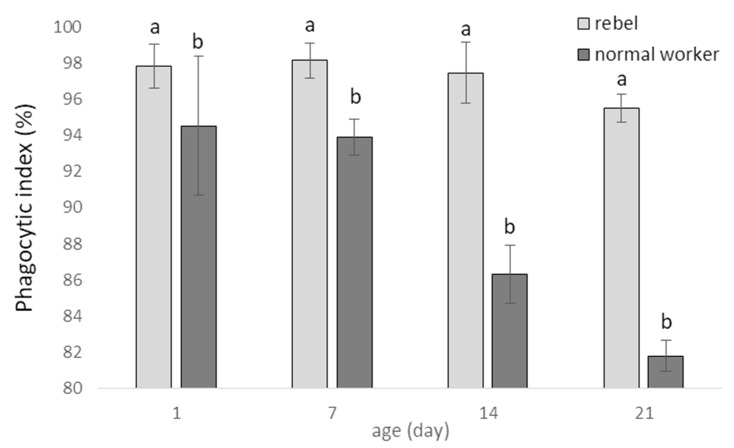
Phagocytic index (%; mean ± SD) in hemolymph of normal workers and rebels. Lowercase letters indicate significant differences between averages for the rebels and normal workers within a specific age with *p* < 0.01; No. of rebels = 60; no. of normal workers = 60.

Phagocytic index (%; mean ± SD) in hemolymph of normal workers and rebels are shown in Figure 2. The phagocytic index decreased with the age in both sub-castes, quicker in normal workers, and was higher in the rebels compared to normal workers in four tested age groups (Figure 2; two-way ANOVA with multiple comparison testing using the post hoc Tukey HSD test; age F_(3,232)_= 248.67; *p* < 0.0001; castes/sub-caste F_(1,232)_= 1265.7; *p* < 0.0001, post-hoc for each group max. *p* = 0.941535; castes/sub-caste*age F_(3,232)_= 124,54, *p* < 0.0001, post-hoc for each group max. *p* = 0.996101; no. of rebels = 60; no. of normal workers = 60).

**Figure 3:**
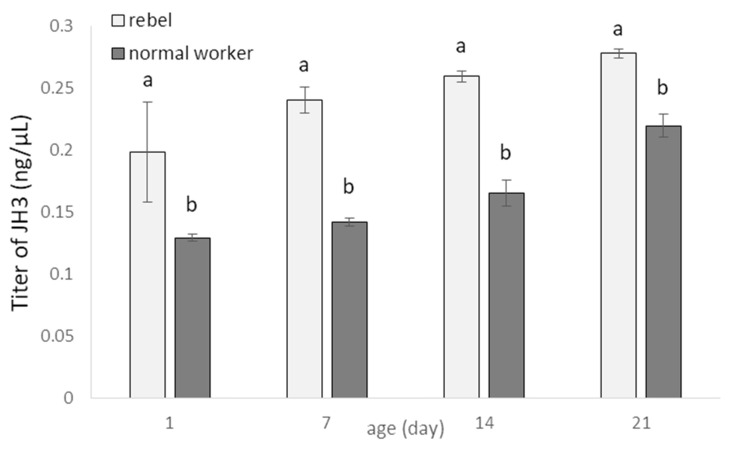
Juvenile hormone (JH3; mean ± SD) titers in hemolymph of normal workers and rebels. Lowercase letters indicate significant differences between castes/sub-caste within a specific age with *p* < 0.01; No. of rebels = 60; no. of normal workers = 60.

JH3 titers increased with age of normal workers and rebels (Figure 3). However, the rebels had higher JH3 levels compared to normal workers (Two-way ANOVA with multiple comparison testing using the post hoc Tukey HSD test; age F_(3,232)_= 307.55, *p* < 0.0001; castes/sub-caste F_(1,232)_= 1531.2; *p* < 0.0001, post-hoc for each group max. *p* < 0.0001; castes/sub-caste*age F_(3,2320)_= 22.550, *p* < 0.0001, post-hoc for each group max. *p* < 0.0399; no. of rebels = 60; no. of normal workers = 60).

**Figure 4:**
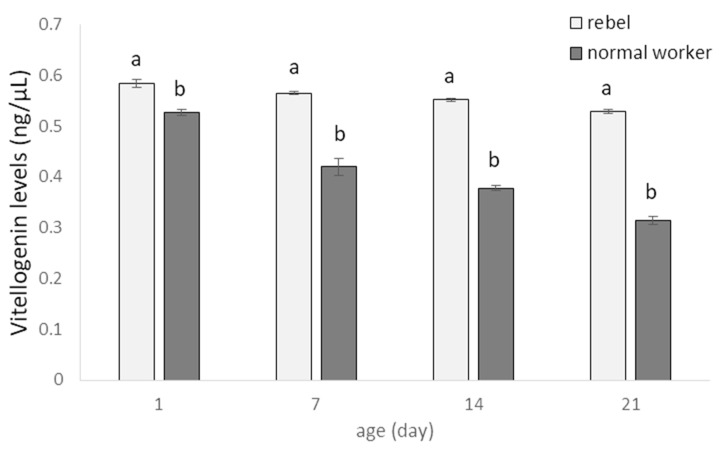
Vitellogenin levels (mean ± SD) in hemolymph of normal workers and rebels. Lowercase letters indicate significant differences between averages for the rebels and normal workers within a specific age with *p* < 0.01; No. of rebels = 60; no. of normal workers = 60.

Vg levels were decreased with ageing, both in rebel and in normal workers (Figure 4), but these values were always higher in rebels regardless of age (Two-way ANOVA with multiple comparison testing using the post hoc Tukey HSD test; age F_(3,2320)_= 2952.0, *p* < 0.0001; castes/sub-caste F_(1,232)_= 20710; *p* < 0.0001, post-hoc for each group max. *p* < 0.0001; castes/sub-caste*age F_(3,232)_= 1058.3, *p* < 0.0001, post-hoc for each group max. *p* = 0.880745; no. of rebels = 60; no. of normal workers = 60).

**Figure 5:**
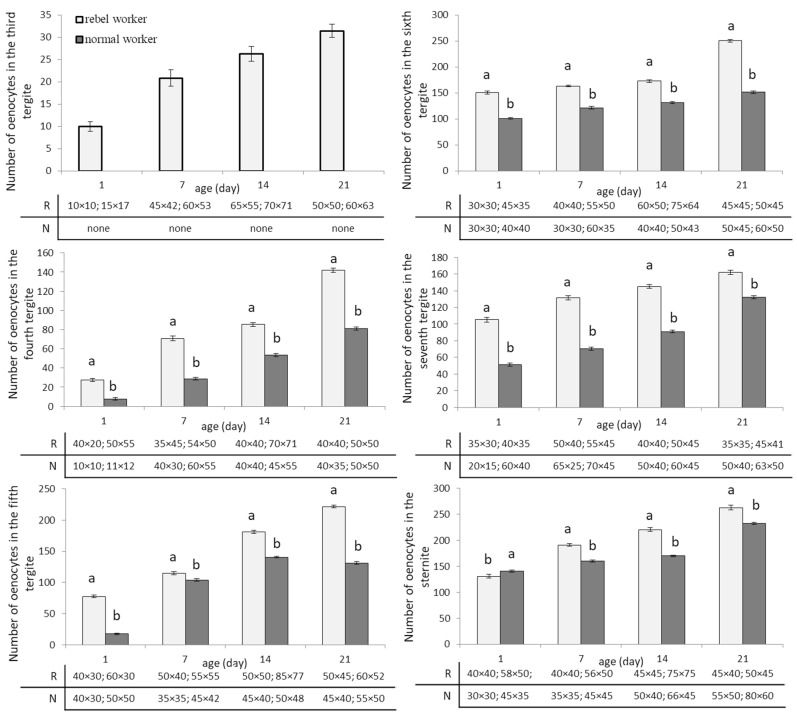
Number and size of oenocytes (mean ± SD) in the fat body from the third–seventh tergites and sternites in aging of rebel and normal workers. The tables below each small graph show the sizes (length × width) of the smallest and largest oenocytes in the fat body of a particular segment. Lowercase letters indicate significant differences between averages for the rebels and normal workers within a specific age with *p* < 0.01; R—rebels; N—normal workers.

The numbers of oenocytes in the fat body of all segments (the fourth, fifth, sixth, and seventh tergite segments and in the sternite) increased with age of the workers and was always higher in rebels (Figure 5; Two-way ANOVA with multiple comparison testing using the post hoc Tukey HSD test; for the third tergite: age F_(3,232)_ = 1080.2; *p* < 0.0001; castes/sub-caste F_(1,232)_ = 25,095.0; *p* < 0.0001, post-hoc for each group max. *p* < 0.0001; castes/sub-caste*age F_(2,232)_ = 1080.2, *p* < 0,0001, post-hoc for each group max. *p* = 0.01; for the fourth tergite: age F_(3,232)_ = 28,103.0; *p* < 0,0001; castes/sub-caste F_(1,232)_ = 27,218.0; *p* < 0.0001; post-hoc for each group max. *p* < 0.0001; castes/sub-caste*age F_(3,232)_ = 1381.2; *p* < 0.0001; post-hoc for each group max. *p* = 0.100453; for the fifth tergite: age F_(3,232)_ = 46,912.0; *p* < 0.0001; castes/sub-caste F_(1,232)_ = 35,353; *p* < 0.0001; post-hoc for each group max. *p* < 0.0001; castes/sub-caste*age F_(3,232)_ = 3836.8; *p* < 0.0001; post-hoc for each group max. *p* < 0.0001; for the sixth tergite: age F_(3,232)_ = 10,128.0; *p* < 0.0001; castes/sub-caste F_(1,232)_ = 32,877.0; *p* < 0.0001; post-hoc for each group max. *p* < 0.0001; castes/sub-caste*age F_(3,232)_ = 1845.9; *p* < 0.0001; post-hoc for each group max. *p* = 0.976218; for the seventh tergite: age F_(3,232)_ = 10,302.0; *p* < 0.0001; castes/sub-caste F_(1,232)_ = 30,316; *p* < 0.0001; post-hoc for each group max. *p* < 0.0001; castes/sub-caste*age F_(3,232)_ = 568.39; *p* < 0.0001; post-hoc for each group max. *p* = 0.988241; for the sternite: age F_(3,232)_ = 14,958.0; *p* < 0.0001; castes/sub-caste F_(1,232)_ = 4427.8; *p* < 0.0001; post-hoc for each group max. *p* < 0.0001; castes/sub-caste*age F_(3,232)_ = 1103.1; *p* < 0.0001; post-hoc for each group max. *p* < 0.0001; no. of rebels = 60; no. of normal workers = 60). In the third tergite segment, oenocytes were not observed during the whole life of normal workers. The smallest number of oenocytes in rebels was observed in one-day-old bees in the third tergite, while in normal workers, at the age of one day in the fourth tergite. The largest number of oenocytes in rebels was seen in 21-day-old bees in the seventh tergite fat body and in normal workers in 21-day-old bees in the sixth tergite. Generally, the number of oenocytes differed between segments increasing from third to sixth but not between the sixth and seventh tergites. In sternite, both in rebels and normal workers, the number of oenocytes increased with age of rebels and normal workers (Figure 5). The smallest oenocytes were observed in one-day-old rebels in the third tergite and in one-day-old normal workers in the fourth tergite. In contrast, the largest oenocytes were seen in 14-day-old rebels in the fifth tergite of the fat body (Figure 5).

## 4. Discussion

Honeybee immunosenescence is not a simple function of chronological age since immunity is linked to the behavioral and physiological role, which in turn can be affected by the social environment [12,31]. So, specific phenotypic specializations are also characterized by different roles [32]. In our case, these specializations were presented by normal workers and rebels of different ages that are bees differing in their life expectancy [33,34]. The phenotypic specialization associated with different tasks is expected to be under contrasting selective forces as a colony might benefit from having sets of workers with highly specialized phenotypes, highly efficient and apt to perform the appointed task. At the same time, specializing may limit task flexibility, therby reducing performance of other tasks when needed [32,35]. Thus, labor division should show an adequate degree of flexibility to allow the colony to rapidly reallocate its resources in response to the environmental demands. This should be the case especially when rebels appear in the colony after a swarm. The rebels have many anatomical and behavioral features distinguishing them from normal workers. Their pheromone economy differs compared to normal workers, and this makes them more queenlike [36]. This difference is the result of changes in the epigenome [37] caused by the lack of queen pheromones in diet during larval development [21]. As a result, compared to normal workers, rebels have a higher level of phagocytosis index, JH and Vg, as well as bigger and more numerous oenocytes. These last parameters included in the humoral and cellular defense cooperate with each other so as to obtain all the desired effects by maintaining homeostasis, and/or eradication of the pathogen [38]. Cellular responses are mediated by hemocytes, the cellular component of hemolymph responsible for nodulation, encapsulation, and phagocytosis of invading pathogens. On the other hand, the hemocytes together with the fat body cells are also involved in the mechanisms of the humoral response that includes AMP synthesis, the enzymatic cascade that regulates the activation of hemolymph coagulation and melanization, and the production of reactive oxygen (ROS) and nitrogen (RNS) species [39]. Hystad et al. [40] suggest that nurses show two-fold higher levels of phagocytic activity compared to foragers. We also observed that young normal workers had a higher phagocytic index than the older bees. On the other hand, the phagocytic index in the rebels remained at a constant level for the first two weeks of their lives and then began to decrease around the 21st day of life. Richardson et al. [41] observed that young queens maintained a large population of hemocytes, mainly plasmatocytes and granulocytes, and therefore they appeared to undergo a slower rate of senescence in immune function. It can be assumed that this is one of the factors that determine the rebel’s longevity [23,37]. The activity of hemocytes depends on the level of Vg which is a zinc donor and plays an important regulatory and catalytic role in the immune system [6]. Vg acts as an opsonin and binds to surface molecules of pathogens, such as lipopolysaccharides [42], which might activate hemocytes. We confirmed that the level of Vg in workers decreases with age [43,44,45,46,47] and can regulate the onset of foraging by interacting with juvenile hormone in a reciprocally inhibitory dynamic such that Vg inhibits juvenile hormone and vice versa [43,44]. The onset of foraging is caused by both a decrease in Vg and/or an increase in JH. Our data are consistent with the presented model since in rebels, similarly as in queens [48], the Vg level was also higher than in the normal workers. These results are also compatible with Lin et al. [49] and Nakaoka et al. [50] who documented that the workers with developed ovaries had higher Vg expression levels than bees with inactivated ovaries. Naeger et al. [51] and Peso et al. [47] added that the higher Vg levels in workers of queenless colonies support ovarian activity and the functioning of hypopharyngeal glands as well as exerting influence on active foraging. In workers, investing in oocyte production before vitellogenesis, when there is no prospect of producing offspring, is wasteful since it is the costliest stage of oogenesis [52,53,54]. But for rebels, when the queen is absent or lost, it is a necessary step, and therefore their Vg levels are higher compared to normal workers. Vg synthesis is stimulated by JH, whose titers were always higher in rebels compared to normal workers of the same age. Corona et al. [9] suggested that JH has higher values in queens than in workers. JH interacts with Vg and the elements of the insulin signaling pathway and regulate queen longevity [9] and most likely that of rebels [23,37]. Pankiw et al. [55] suggested that the presence of a queen and/or her mandibular gland pheromone (QMP) inhibit the JH synthesis. So, a social insect queen pheromone can act as a modulator for labor division. Therefore, non-reproductive females have lower values of JH. Moreover, JH affects the development of oenocytes [5]. In turn, oenocytes secrete ecdysteroids that regulate several functions during development and insect reproduction [56,57] through the production of the outer layer of egg chorion [58]. As it was mentioned, JH titer in the workers increases with age, while that of ecdysteroids decreases [59]. However, egg-laying workers have high titers of ecdysteroids. These hormonal changes have an influence on oenocytes [5,59], whose number increased with age of bees. This may also explain why the numbers of oenocytes were always higher in rebels than in normal workers. These cells were typically oval, round, or elongated in shape in each segment of the subcuticular fat body. The length and width of these cells were higher in rebels and their values were increased until the 14th day of their lives and next, decreased. Koubová et al. [59] observed that the highest diameters of oenocytes occur in December and January. It follows that the size of oenocytes depends on seasonal changes being the result of the physiological condition and phenotype of the individual. Moreover, Paes-de-Oliveira et al. [5] suggested that the extra dose of the JH stimulated to the increase of oenocyte size. This may be one of the explanations why we saw larger cells in the rebels than in normal workers. The application of the JH alters the physiology of workers to conditions similar to those observed in queens [5]. Reducing the size of oenocytes in older bees may also be associated with a reduction of JH titers. Moreover, as suggested by Koubová et al. [59], this decreased fat body cell size is associated with a lower rate of DNA synthesis and lower telomerase activity. These changes result in the release of energy reserves contained in the cells [4,5]. We also confirmed that oenocytes located in the fat body in the third tergite did not occur in normal worker bees, only in the females with an increased reproductive potential [4]. Previous research by Strachecka et al. [4] showed that the fat body is segmental. The present paper analyses further parameters (e.g., the numbers and sizes of oenocytes in specific segments/localizations of this tissue). Their presence in the rebels and queens indicate an adaptation to the performance of the reproductive function in comparison with sterile females/normal workers.

## 5. Conclusions

The physiological state of an insect depends on the efficiently functioning immune mechanisms (i.e., cellular and humoral defenses). Rebels develop in a queenless colony, can lay eggs, maintain tasks inside and outside the colony, and thus exhibit traits both of a queen and a worker. Our research shows that, firstly, rebels are characterized by a higher phagocytic index compared to normal workers, which demonstrates more efficient defense mechanisms at the cellular level. Secondly, they have higher levels of JH and Vg, which confirms their higher reproductive potential and the ability to lay eggs. Moreover, higher concentrations of JH stimulate an increase in the size of oenocytes, which we also confirmed in our research. And thirdly, the greater numbers and sizes of oenocytes, which are responsible for hormonal regulation mainly in reproductive females (predominantly queens), are morphological adaptations of the rebels to life in the eusocial society of honeybees. It can therefore be concluded that the reproductive dominance of the so-called rebel workers is the result of anatomical (e.g., the number of ovarioles), morphological (in the fat body), and biochemical (in the hemolymph) changes.

## Data Availability

The datasets generated during and/or analyzed during the current study are available from the corresponding author on reasonable request.
